# Larvae of the Clothing Moth *Tineola bisselliella* Maintain Gut Bacteria that Secrete Enzyme Cocktails to Facilitate the Digestion of Keratin

**DOI:** 10.3390/microorganisms8091415

**Published:** 2020-09-14

**Authors:** Andreas Vilcinskas, Michael Schwabe, Karina Brinkrolf, Rudy Plarre, Natalie Wielsch, Heiko Vogel

**Affiliations:** 1 Institute for Insect Biotechnology, Justus-Liebig-University of Giessen, Heinrich-Buff-Ring 26-32, 35392 Giessen, Germany; Andreas.Vilcinskas@ime.fraunhofer.de; 2 Department of Bioresources, Fraunhofer Institute for Molecular Biology and Applied Ecology, Ohlebergsweg 12, 35392 Giessen, Germany; michael.schwabe@ime.fraunhofer.de (M.S.); karina.brinkrolf@bio.uni-giessen.de (K.B.); 3 Biologische Materialschädigung und Referenzorganismen, Bundesanstalt für Materialforschung und -prüfung (BAM), Unter den Eichen 87, 12205 Berlin, Germany; Ruediger.Plarre@bam.de; 4 Research Group Mass Spectrometry/Proteomics, Max-Planck Institute for Chemical Ecology, Hans-Knoell-Strasse 8, 07745 Jena, Germany; nwielsch@ice.mpg.de; 5 Entomology Department, Max-Planck Institute for Chemical Ecology, Hans-Knoell-Strasse 8, 07745 Jena, Germany

**Keywords:** insect biotechnology, beneficial microbes, symbiosis, keratin, dietary adaptation, Tineola bisselliella

## Abstract

The evolutionary success of insects is promoted by their association with beneficial microbes that enable the utilization of unusual diets. The synanthropic clothing moth *Tineola bisselliella* provides an intriguing example of this phenomenon. The caterpillars of this species have adapted to feed on keratin-rich diets such as feathers and wool, which cannot be digested by most other animals and are resistant to common digestive enzymes. Inspired by the hypothesis that this ability may be conferred by symbiotic microbes, we utilized a simple assay to detect keratinase activity and a method to screen gut bacteria for candidate enzymes, which were isolated from feather-fed larvae. The isolation of DNA from keratin-degrading bacterial strains followed by de novo genome sequencing resulted in the identification of a novel bacterial strain related to *Bacillus* sp. FDAARGOS_235. Genome annotation identified 20 genes with keratinase domains. Proteomic analysis of the culture supernatant from this gut bacterium grown in non-nutrient buffer supplemented with feathers revealed several candidate enzymes potentially responsible for keratin degradation, including a thiol-disulfide oxidoreductase and multiple proteases. Our results suggest that the unusual diet of *T. bisselliella* larvae promotes their association with keratinolytic microorganisms and that the ability of larvae to feed on keratin can at least partially be attributed to bacteria that produce a cocktail of keratin-degrading enzymes.

## 1. Introduction

In terms of species numbers, insects are the most diverse group of organisms on earth. Their evolutionary success has in part been attributed to their ability to manage associated beneficial microbes in order to utilize unusual diets [1]. Insect-derived microbes are therefore recognized as a bioresource to be explored for human welfare, e.g., for the bioconversion of organic waste [2]. The clothing moth *Tineola bisselliella* is a global synanthropic pest because the caterpillars can feed on clothes and carpets made of wool [3]. Autecological, behavioral, and historical data combined with published faunistic records suggest that this species probably originated from Central or Southern Africa and was introduced into Europe in the late 18th century. Its preference for dry environments promoted its worldwide spread during the 20th century when indoor climates changed because of the introduction of domestic central heating systems [3]. *T. bisselliella* has adapted to feed predominantly on materials rich in keratin, such as feathers, woolen clothes, and carpets. Keratin is particularly resistant to proteolytic degradation because of its abundant disulfide bonds (cysteine content = 7–13%), which distinguishes it from other structural proteins such as collagen and elastin [4]. Only sophisticated microbes can degrade keratin [5]. A subtractive library of transcripts from the gut of *T. bisselliella* larvae identified serine-like and chymotrypsin-like proteases as candidate keratinases, but neither cysteine-like proteases nor metalloproteinases were detected [6]. To date, there are no candidate keratinase enzymes reported, which could mediate keratin digestion in the gut of *T. bisselliella.*

Given that many insects are associated with beneficial microbes providing enzymes which are essential for the adaptation to unusual diets, we postulated that microbes could play a similar role in *T. bisselliella*. To determine whether the ability of *T. bisselliella* larvae to utilize keratin is assisted by its gut microbiome, we isolated bacteria from the guts of caterpillars fed on a diet of feathers as a sole nutrient source. The culture supernatants were screened for keratinase activity using a simple photometric assay and the most promising strains were analyzed in more detail. Here, we report the identification of novel *Bacillus* strains found in the *T. bisselliella* larval gut with the apparent ability to digest keratin. Cultivation of these strains on sterilized feathers followed by proteomic analysis of the culture supernatant resulted in the identification of complex mixtures of enzymes and other proteins that could contribute to the digestion of keratin by *T. bisselliella* caterpillars.

## 2. Materials and Methods

### 2.1. Insect Rearing and Bacterial Isolation

**Figure 1:**
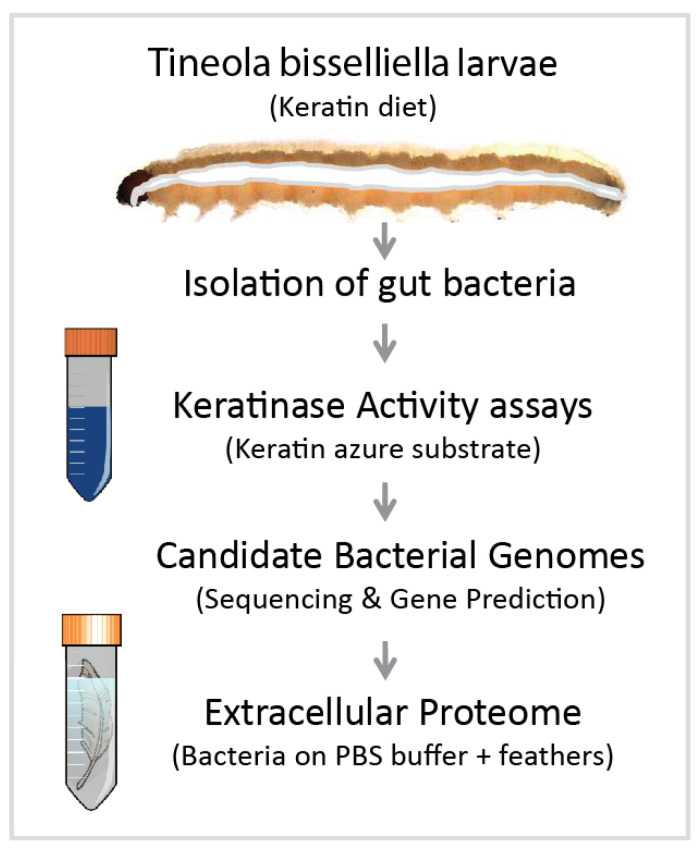
Experimental workflow for the identification of keratin-degrading proteins in bacteria associated with the clothing moth *Tineola bisselliella*. Clothing moth larvae were reared on a keratin-based diet for several generations before the isolation of gut bacteria. Individual bacterial colonies were then tested for keratinase activity using keratin azure as a substrate. DNA was extracted from two bacterial isolates showing the strongest activity in the keratin azure assay as well as the ability to grow on whole feathers as a sole nutrient source. The genome sequence was screened for protein-coding genes allowing the identification of candidate keratinases. Extracellular proteins isolated from the supernatant of bacteria grown on feathers as a sole nutrient source were analyzed by LC MS-MS.

**Figure 2:**
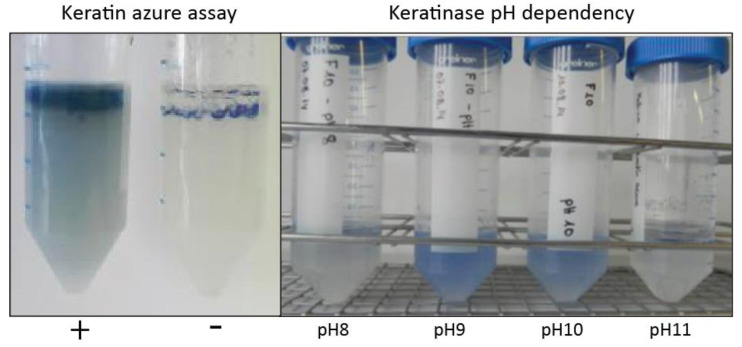
Keratin azure assay for bacterial strain keratinase activity. Individual bacterial colonies were inoculated in PBS buffer with the substrate keratin azure (blue-colored sheep wool) to allow the photometric quantification of keratinase activity, which releases a blue dye into the supernatant (left panel). Addition of bacterial culture medium without bacteria was used as a negative control (−). Addition of bacterial culture medium with highest keratinolytic activity was used as a positive control (+). Since keratinolysis is pH dependent, all isolates were tested in buffers with a pH range of 6–12 (right panel).

Specimens of *T. bisselliella* were obtained from the Federal Institute for Materials Research and Testing (Berlin, Germany) and were reared on an exclusive diet of feathers at room temperature. The feathers were surface sterilized with 80% ethanol to kill attached bacteria before feeding. Caterpillars were surface sterilized before dissection and the extraction of gut bacteria, which was achieved by macerating the entire gut in sterile phosphate-buffered saline (PBS) and preparing serial dilutions of the homogenate. Samples were taken from different regions of the gut. Isolates were tested for general proteolytic activity on casein plates, and positive isolates were cultivated with a keratin azure substrate (blue-colored sheep wool) in PBS (37 °C) to allow the photometric (A 595 nm) quantification of keratinase activity, which releases a blue dye into the supernatant (Figure 1 and Figure 2).

One unit of keratinase activity was defined as an increase in absorbance of 0.01 when comparing the sample to the PBS control. Keratinolysis is pH dependent, so we tested all isolates in buffers with a pH range of 6–12 (Figure 2). The isolates with the highest keratinolytic activity were selected for genomic analysis. The experimental keratinase activity quantification was repeated two times with three falcon tubes per tested pH-value.

### 2.2. Genome Sequencing and Characterization of Bac18 Isolated from T. bisselliella

Sequencing was carried out at the Max Planck Genome Center (Cologne, Germany) using technology of a Pacific Biosciences Sequel instrument. A total of 90,570 PacBio raw reads were assembled with HGAP3 (Hierarchical Genome Assembly Process 3) [7] with default options and an estimated genome size of 5 Mb. We checked for circular contigs using Circlator 1.5.1 [8] in “all” mode using the corrected reads from the HGAP3 pipeline. Genome annotation was carried out using results from Circlator for Prokka 1.11 [9] in default mode with “Bacteria” as kingdom. Nucleotide sequence alignments of the 16S rRNA genes copies were aligned with Clustal X [10]. The closest reference genomes (fully sequenced and assembled) were estimated using ReferenceSeeker 1.10 [11] in default mode with the implemented database “bacteria” (August 2019). Bac18 was analyzed for its global sequence identity to ReferenceSeeker’s best hit with the MUMmer3 package [12].

### 2.3. Target Gene Prediction

For the detection of genes involved in keratin degradation, UniProt (The UniProt Consortium, 2019, date: 29 August 2019) was searched for the keyword “keratinase” and the resulting FASTA (canonical) and associated text files were downloaded. Non-keratinase entries (A0A1S6Y3A5, A0A0G3BBA5) were manually identified and removed before further analysis. Pfam [13] domains were extracted from the corrected UniProt text file. In a second step, the coding sequences in Bac18 (predicted using Prokka) were compared to the PfamA-31 database using the commands hmmscan --domtblout, --noali, and --notextw in HMMER v3.1b2. The corresponding Pfam annotations were searched for keratinase domains and classified using an in-house script. In the last step, we performed a local alignment of all coding sequences and keratinase results from UniProt using BLASTp in BLAST v2.6.0+ [14].

### 2.4. Proteomic Analysis

Extracellular bacterial proteins contained in feather-supplemented media were concentrated using the Vivaspin™ ultrafiltration spin columns (GE Healthcare Life Sciences) according to the manufacturers’ recommendations. Protein mixtures were subsequently separated by sodium dodecylsulfate polyacrylamide gel electrophoresis (SDS-PAGE) on 4–12% Criterion XT gradient gels (BioRad, Hercules, CA, USA) with XT MES running buffer at 125 V for 1.5 h. The molecular weights (kDa) of the separated proteins were estimated using pre-stained markers and unstained high-mass-precision protein markers. For LC-MS/MS analysis, protein bands were excised from the gel as 10 molecular weight blocks per lane, followed by tryptic digestion as described by [15]. LC-MS sample processing, data acquisition, and data processing were carried out as previously described [16]. MS BLAST was then used to search a database derived from the in silico translation of the Bac18 genome. Search parameters specifying mass measurement accuracy, minimum number of product ion matches per peptide, minimum number of product ion matches per protein, minimum number of peptide matches, and maximum number of missed tryptic cleavage sites are detailed in the Supplementary Materials.

## 3. Results

### 3.1. Identification of Bacterial Candidates and Genome Sequencing

Keratin-degrading bacteria and candidate keratinases in the *T. bisselliella* larval gut were identified by following the scheme shown in Figure 1. First, we dissected the guts of *T. bisselliella* larvae feeding on an exclusive keratin-based diet and cultivated serially diluted gut homogenates on casein to identify colonies with extracellular proteolytic activity. Individual colonies were then inoculated into the substrate keratin azure, which produces an easily detected blue dye when digested (Figure 2). We also tested for keratinolytic activity by incubating bacteria in a non-nutrient buffer containing feathers, which began to fragment in the presence of keratinase activity. Based on these results, we selected individual bacterial colonies with the highest overall keratinolytic activity for genome sequencing. Alignment of the 16S rRNA genes indicated that the selected bacterial isolates represented the same species and strain. We therefore focused on one of the two *Bacillus* candidates (Bac18) for further genome analysis, proteomics, and keratinase assays.

The final draft genome of Bac18 is represented by one circular chromosomal genome with a size of 5.2 Mb and additional five small contigs with a total length of 827 kb (Table 1). We identified 5946 protein coding sequences, 14 rRNA gene clusters, and 106 tRNA genes. A nucleotide sequence alignment of the 16S rRNA gene copies led to the identification of eight different variants that differ from each other by SNPs at six variable positions. The closest relative on the whole genome level was calculated using all publicly available genomes that are fully sequenced and assembled (Table 2). This calculation includes small contigs of Bac18 as well as potential plasmid sequences of the references. The conserved DNA is the fraction of both genomes that are homologous while the ANI is the average nucleotide identity of this homologous region.

**Figure 3:**
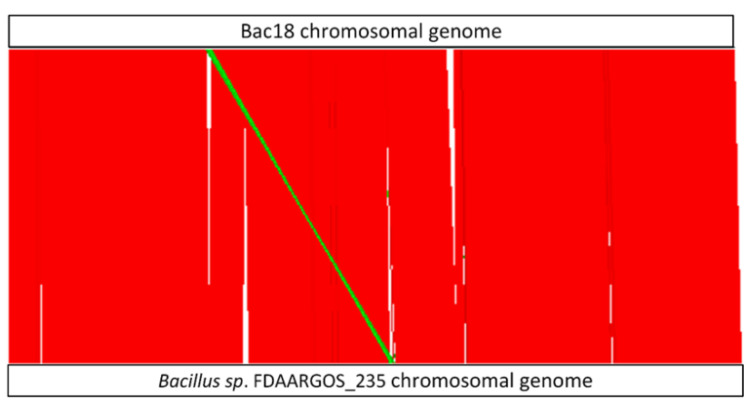
Visualization of the structural similarities of Bac18 and *Bacillus* sp. FDAARGOS_235 chromosomal genomes calculated with Nucmer. Genomes are given as white boxes at the top (Bac18) and the bottom (*Bacillus* sp.) of the illustration. For comparison, both circular genomes are opened at the starting position of the *dnaA* gene. Red: homologous sequences with the same orientation; green: reverse complement, but homologous sequences, white: regions without homologies.

We found that Bac18 and *Bacillus* sp. FDAARGOS_235 share 83.93% of their genomes (conserved DNA) with an overall average nucleotide identity (ANI) of 99.56%. In this regard it has to be noted that the small contigs of Bac18 make up ~14% of the length of the genome. A visualization of the structural similarities of the Bac18 chromosomal sequence with *Bacillus* sp. FDAARGOS_235 is shown in Figure 3, where homologous regions between both genomes are represented in red (homologous sequences with same orientation) and green (reverse complement, but homologous region). No such whole-contig similarities were detected for the respective small contigs of Bac18 with the plasmids of the closest reference. Nevertheless, we were able to detect homologies of fragments of the small Bac18 contigs with publicly available plasmid sequences using BLASTn analysis. However, none of these plasmids were covered completely.

### 3.2. Candidate Gene Prediction and Proteomic Analysis

**Figure 4:**
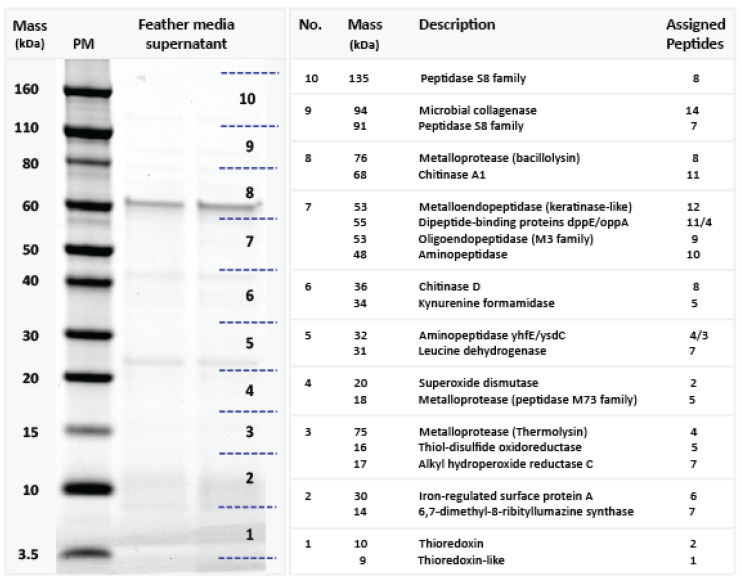
Analysis of extracellular proteins isolated from the supernatant of Bac18 growing on feathers as a sole nutrient source. Left panel shows SDS-PAGE protein gel divided into 10 zones with different molecular weight ranges (PM = protein size markers). Right panel shows the properties of the identified proteins, including predicted masses, descriptions, and the number of assigned peptides. The matching predicted proteins in the bacterial genome are listed in electronic Supplementary Materials, File S1.

We searched the Bac18 genome for potential keratinase-encoding genes and identified 20 sequences with predicted keratinase domains as found in Pfam. The amino acid sequences of the 20 candidates were used as BLAST queries against keratinase entries in UniProt. Table 3 shows the best hits against UniProt filtered for sequence coverage (≥70%) and sequence identity (≥40%). With a sequence coverage of 100% and a sequence identity of 98% the gene PROKKA_03102 has the highest blast score against a keratinase included in UniProt. The supernatant from the non-nutrient medium containing fragmented feathers was collected after Bac18 had been growing for 72 h, allowing us to identify candidate extracellular proteins with keratinase activity. The concentrated extracellular proteins were separated by SDS-PAGE and bands were observed with apparent molecular masses ranging from <5 kDa to >100 kDa (Figure 4). The gel lanes were divided into ten blocks covering different size ranges for in-gel digestion with trypsin. The resulting peptides were then analyzed by LC-MS/MS using protein sequences predicted in silico from the Bac18 genome as a database.

We identified 63 different proteins in the feather-supplemented culture supernatant that matched sequences predicted in the Bac18 genome using our proteomics cut-off criteria (Supplementary Materials, File S1). We subsequently focused on those candidates for which there was evidence for direct or indirect involvement in keratin utilization. The largest group of these proteins was annotated as proteases. We identified 16 different proteases, including serine-type and metalloproteinases, with predicted protein masses of 18–135 kDa (Figure 4). Of particular interest were candidate proteases related to previously characterized bacterial keratinases, such as specific extracellular metalloproteases and the subtilisin family of serine proteases. We also identified a thiol-disulfide oxidoreductase, which is likely to facilitate the proteolytic degradation of keratin by promoting the hydrolysis of disulfide bonds.

## 4. Discussion

Caterpillars of the clothing moth *T. bisselliella* feed predominantly on keratin-based materials such as feathers, hairs, and wool. The midgut of these insects is anaerobic with a negative redox potential, which was proposed to facilitate the solubilization of keratin by reducing disulfide bonds, enabling digestion by more widespread proteases [17]. However, even in a high redox potential environment, only a subset of proteases can degrade keratin substrates [18]. Analysis of the crude gut preparations resulted in identification of aminopeptidase activity and a l-Cysteine lyase, but neither cysteine endopeptidase nor metalloprotease activities were found [19,20]. Although multiple serine proteases with high expression levels were identified in the *T. bisselliella* gut transcriptome [6], their involvement in keratin proteolysis is unclear.

Therefore, it remains to be determined whether serine proteases encoded by the host insect genome can process keratin, or whether additional enzymatic or physiological functions are required to initiate the degradation of this molecule by reducing the abundant disulfide bonds. 

An alternative explanation for the adaptation of the clothing moth to keratin-based diets is that keratinase activity is conferred by specific bacterial symbionts in the gut. Although previous studies found no abundant microorganisms in *T. bisselliella* larvae [21,22], we were able to reproducibly isolate bacteria from the midgut of larvae fed on a feather diet. Most of the isolates were identified as *Clostridiales*, *Lactobacillales,* and the genus *Bacillus*, similar to the bacteria associated with the brown house moth *Hofmannophila pseudospretella*, another keratin-feeding lepidopteran [23]. After screening individual gut-isolated bacterial strains for their ability to degrade keratin, we selected two isolates showing the highest levels of keratinase activity in both the keratin azure and whole-feather assays. These were found to represent the same species, and genomic sequencing of isolate Bac18 identified a new *Bacillus* strain with a close relationship to *Bacillus* sp. FDAARGOS_235 and *B. thuringiensis*. Interestingly, *Bacillus* species are predominant among bacteria displaying keratinase activity, and are known to encode for several keratin-degrading proteases.

On a keratin diet, larvae of the clothing moth develop very slowly, possibly owing to the limited availability of an easily accessible nitrogen source. Likewise, in our assays using non-nutrient buffer supplemented with whole feathers, the inoculated gut-associated bacteria showed slow growth. When clothing moth larvae or their gut-associated bacteria were provided solely with keratin (whole feathers in our experiments) as a nitrogen source, we hypothesized that nitrogen assimilation mechanisms are induced, including the expression and secretion of keratin-active enzymes [24,25,26], which subsequently requires polypeptide processing and active transport. Accordingly, proteomic analysis of culture supernatants from Bac18 grown on non-nutrient medium supplemented with feathers revealed the presence of enzymes with the potential to degrade keratin, including collagenases and other serine proteases, metalloproteases, and a thiol-disulfide oxidoreductase. These findings support our hypothesis that the unusual diet of *T. bisselliella* larvae promotes their association with keratinolytic microorganisms and that keratin digestion is potentially supported by bacteria in the larval gut. The thiol-disulfide oxidoreductase was particularly interesting because this enzyme can hydrolyze disulfide bonds, thus enabling the complete proteolytic degradation of keratin and subsequent nitrogen assimilation [25,26].

We detected a surprisingly complex mixture of numerous extracellular proteases as well as enzymes that modify bacterial cell walls, peptide-binding proteins, transporters, and stress-response factors. These functions are commensurate with the stress anticipated when bacteria are forced to grow on keratin as a sole nutrient source. Previous studies of bacterial keratinases reviewed in [18,19] have focused on a single enzyme while ignoring the complexity of the extracellular proteome. The presence of such a rich cocktail of extracellular proteins suggests that the combinatorial action of multiple enzymes, transporters, and stress-response proteins ensures the more efficient degradation of keratin compared to a single, isolated protease. This hypothesis is supported by previous reports that certain *Bacillus* species encode multiple proteases representing different functional classes and different protein masses that nevertheless possess keratinase activity [27,28].

Based on the proteins detected in supernatants of Bac18 cultivated in nutrient-free medium supplemented with feathers, we propose that keratin digestion is a multistep process beginning with the cleavage of disulfide bonds by a thiol-disulfide oxidoreductase, enhancing the accessibility of the reduced keratin polypeptide for a cocktail of exopeptidases (aminopeptidases and dipeptidyl peptidases) [22,28] and endo-active proteases such as subtilisins, collagenases, and other metalloendoproteases, and oligoendopeptidases. These digest keratin into shorter peptides and free amino acids, which are sequestered by transport proteins and assimilated. Our results do not rule out the possibility that *T. bisselliella* also produces its own enzymes that contribute to keratin digestion, but such native keratinases have yet to be identified [6]. The isolated keratin-degrading bacteria help to explain the ability of *T. bisselliella* to occupy a unique ecological niche. Strikingly, the tarantula *Chilobrachys guangxiensis,* which requires powerful enzymes to digest the immobilized preys down into a liquid taken up with straw-like mouthparts into the intestines, was also found to harbor feather-degrading bacteria in the gut producing keratinases [29]. Such keratinase-producing bacteria are of interest for biotechnological applications, particularly in the bioconversion of keratin-rich waste such as feathers, hair and woolen textiles [2]. Further, we expanded the number of bacteria associated with moth pest insects [30]. 

## Figures and Tables

**Table 1:** Assembly statistics for Bac18. Circularized contigs are marked with •.

Bac18		
Contig Name	Contig Length [bp]	Sequencing Coverage
Bac18_0	• 5,236,241	131
Bac18_2	745,926	164
Bac18_3	• 16,484	479
Bac18_4	39,074	365
Bac18_5	12,019	167
Bac18_6	14,098	173
	∑ 6,063,842	

**Table 2:** Closest reference genome for Bac18 calculated with ReferenceSeeker.

	ID Related Genome	ANI	Cons. DNA	Taxonomy ID	Organism Name
**Bac18**	GCF_002073415.2	99.56%	83.93%	1839798	*Bacillus* sp. FDAARGOS_235
	GCF_000300475.1	98.54%	84.50%	1195464	*Bacillus thuringiensis* MC28
	GCF_000496285.1	99.44%	78.04%	1415784	*Bacillus toyonensis* BCT-7112

**Table 3:** Predicted genes of Bac18 that include Pfam domains related to keratinases.

Prokka ID	Prokka Annotation	Pfam Domains	Keratinase Blast Hit	Proteomics ID
PROKKA_00129	Aminopeptidase YwaD precursor	PA, Peptidase_M28	n. h.	02318
PROKKA_00135	Thermolysin precursor	FTP, Peptidase_M4_C, Peptidase_M4	n. h.	02311
PROKKA_00413	Neutral protease B precursor	FTP, PepSY, Peptidase_M4, Peptidase_M4_C	A0A0B4ZU77	02032
PROKKA_00751	Peptidase propeptide and YPEB domain protein	PepSY	n. h.	01721
PROKKA_01081	Minor extracellular protease vpr precursor	Peptidase_S8, Inhibitor_I9, PA	n. h.	01389
PROKKA_01290	Peptidase T	Peptidase_M28 *	n. h.	01180
PROKKA_01747	Microbial collagenase precursor	Peptidase_S8 *, PPC	n. h.	00731
PROKKA_02134	Bacillolysin precursor	FTP, PepSY, Peptidase_M4, Peptidase_M4_C	n. h.	00344
PROKKA_02729	Bacillolysin precursor	FTP, PepSY, Peptidase_M4, Peptidase_M4_C	n. h.	
PROKKA_02930	Thermolysin precursor	FTP, PepSY, Peptidase_M4, Peptidase_M4_C	n. h.	
PROKKA_03102	Thermitase	Peptidase_S8	F8SVT0 **	
PROKKA_03248	Bacillolysin precursor	FTP *, PepSY	n. h.	02311
PROKKA_03479	Intracellular serine protease	Peptidase_S8	Q45GC8	
PROKKA_04411	hypothetical protein	PepSY	n. h.	03334
PROKKA_04743	Minor extracellular protease vpr	Peptidase_S8, Inhibitor_I9, PA	n. h.	03020
PROKKA_04780	Bacillolysin precursor	FTP, PepSY, Peptidase_M4, Peptidase_M4_C	A0A0B4ZU77	02981
PROKKA_05541	Major intracellular serine protease	Peptidase_S8 *	n. h.	
PROKKA_05686	Neutral protease B precursor	FTP, PepSY, Peptidase_M4, Peptidase_M4_C	A0A0B4ZU77	02032
PROKKA_05790	Thermophilic serine proteinase	Peptidase_S8 *	n. h.	
PROKKA_05803	Chitinase A1 precursor	Peptidase_S8 *	n. h.	

* Pfam domain is not complete (domain coverage < 90%); ** Best BLAST hit; n. h.: no hit.

## Supplementary Materials

The following are available online at https://www.mdpi.com/2076-2607/8/9/1415/s1, File S1: MSE-based Identification of Proteins.
